# TRP Channel Involvement in Salivary Glands—Some Good, Some Bad

**DOI:** 10.3390/cells7070074

**Published:** 2018-07-11

**Authors:** Xibao Liu, Hwei Ling Ong, Indu Ambudkar

**Affiliations:** Secretory Physiology Section, National Institute of Dental Research, National Institutes of Health, Bethesda, MD 20892, USA; Xiliu@mail.nih.gov (X.L.); ongh@mail.nih.gov (H.L.O.)

**Keywords:** TRP channels, calcium signaling, salivary glands, xerostomia, radiation, inflammation

## Abstract

Salivary glands secrete saliva, a mixture of proteins and fluids, which plays an extremely important role in the maintenance of oral health. Loss of salivary secretion causes a dry mouth condition, xerostomia, which has numerous deleterious consequences including opportunistic infections within the oral cavity, difficulties in eating and swallowing food, and problems with speech. Secretion of fluid by salivary glands is stimulated by activation of specific receptors on acinar cell plasma membrane and is mediated by an increase in cytosolic [Ca^2+^] ([Ca^2+^]_i_). The increase in [Ca^2+^]_i_ regulates a number of ion channels and transporters that are required for establishing an osmotic gradient that drives water flow via aquaporin water channels in the apical membrane. The Store-Operated Ca^2+^ Entry (SOCE) mechanism, which is regulated in response to depletion of ER-Ca^2+^, determines the sustained [Ca^2+^]_i_ increase required for prolonged fluid secretion. Core components of SOCE in salivary gland acinar cells are Orai1 and STIM1. In addition, TRPC1 is a major and non-redundant contributor to SOCE and fluid secretion in salivary gland acinar and ductal cells. Other TRP channels that contribute to salivary flow are TRPC3 and TRPV4, while presence of others, including TRPM8, TRPA1, TRPV1, and TRPV3, have been identified in the gland. Loss of salivary gland function leads to dry mouth conditions, or xerostomia, which is clinically seen in patients who have undergone radiation treatment for head-and-neck cancers, and those with the autoimmune exocrinopathy, Sjögren’s syndrome (pSS). TRPM2 is a unique TRP channel that acts as a sensor for intracellular ROS. We will discuss recent studies reported by us that demonstrate a key role for TRPM2 in radiation-induced salivary gland dysfunction. Further, there is increasing evidence that TRPM2 might be involved in inflammatory processes. These interesting findings point to the possible involvement of TRPM2 in Sjögren’s Syndrome, although further studies will be required to identify the exact role of TRPM2 in this disease.

## 1. Introduction

**Figure 1:**
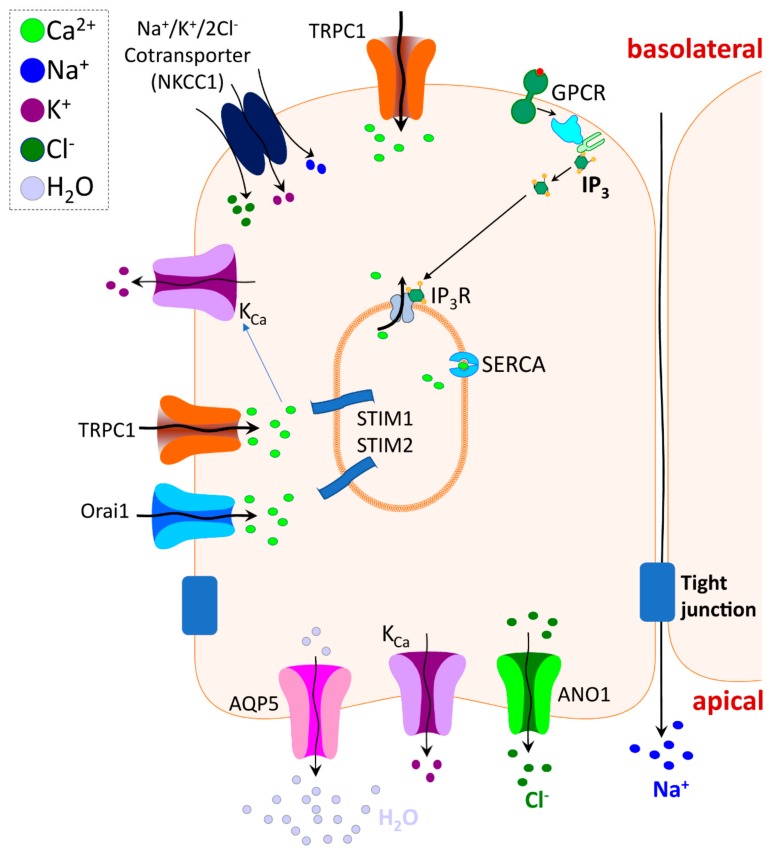
Ca^2+^ signaling and ion channel regulation underlying salivary gland fluid secretion. Salivary gland acinar cell (depicted in the figure) secretes fluid composed of water and electrolytes in response to neurotransmitter stimulation of plasma membrane receptors and consequent elevation of cytosolic [Ca^2+^] ([Ca^2+^]_i_) (see description in the text).

Salivary glands secrete fluid composed of water and electrolytes in response to neurotransmitter stimulation of plasma membrane receptors that cause an elevation of cytosolic [Ca^2+^] ([Ca^2+^]_i_) in acinar cells, which are the primary site of fluid secretion [1,2,3] (Figure 1). The [Ca^2+^]_i_ increase is initiated by stimulation of the major receptors regulating fluid secretion, such as muscarinic cholinergic and α_1_-adrenergic receptors, which triggers activation of phospholipase C (PLC), phosphatidylinositol 4,5-bisphosphate (PIP_2_) hydrolysis, generation of inositol 1,4,5, trisphosphate (IP_3_), and release of Ca^2+^ from the endoplasmic reticulum (ER) Ca^2+^ stores, mediated via the IP_3_ receptors (IP_3_R). In the absence of extracellular Ca^2+^, release from the ER causes a transient increase in [Ca^2+^]_i_ that is not sufficient to maintain prolonged fluid secretion. The latter requires sustained increases in [Ca^2+^]_i_ that is supported by Ca^2+^ influx into the cells. The primary function of the [Ca^2+^]_i_ increase is to regulate the function of ion transporters and channels such as Na^+^/K^+^/2Cl^−^ cotransporter 1 (NKCC1), Anoctamin 1 (ANO1), and Ca^2+^-dependent K^+^ (K_Ca_), which cause vectorial transport of Cl^−^ from the basolateral to the luminal side of the cell, and the generation of an osmotic gradient across the luminal membrane of the cell. The latter provides the driving force for water secretion through the apical membrane via the water channel, Aquaporin 5 (AQP5).

In salivary gland acinar cells, IP_3_-mediated Ca^2+^ release occurs primarily via IP_3_R2 and -3 [4,5]. Importantly, the resulting decrease in ER-[Ca^2+^] triggers the activation of Ca^2+^ influx. This type of Ca^2+^ entry, termed store-operated Ca^2+^ entry (SOCE), provides critical Ca^2+^ signals for regulation of salivary fluid secretion [6,7,8]. SOCE has two types of components; (i) plasma membrane Ca^2+^ channels and (ii) regulatory proteins that sense the change in ER-[Ca^2+^] and gate the channels. The main Ca^2+^ channels involved in SOCE in salivary gland cells are Orai1 and TRPC1. STIM1, an ER-Ca^2+^ binding protein, functions as the ER-[Ca^2+^] sensor and the gating component of both these channels [9,10,11,12,13,14,15]. In addition, STIM2 also contributes to SOCE by enhancing the sensitivity of SOCE activation under conditions when ER-Ca^2+^ stores are not substantially depleted [16]. Several different studies have demonstrated that TRPC1 is an essential channel for salivary gland function, where loss of the channel causes significant loss of fluid secretion and SOCE [17,18,19]. While Orai1 has been extensively studied, its exact role in salivary gland function has not yet been established. One possible function of Orai1 in the gland could be to regulate TRPC1 function, since studies with salivary gland cell lines have demonstrated that TRPC1 function is completely dependent on Orai1 [20,21,22,23].

While [Ca^2+^]_i_ increases are essential for the regulation of salivary gland function, disruption of Ca^2+^ homeostasis, either in resting or stimulated cells, results in salivary gland dysfunction. Two major conditions result in loss of salivary gland function and tissue damage: primary Sjögren’s syndrome (pSS), a chronic autoimmune disease involving lymphocytic infiltration and loss of secretory function in salivary and lacrimal glands [24,25], and radiation-induced salivary gland dysfunction. Radiation-induced xerostomia, or dry mouth condition, occurs in patients who undergo radiation therapy for head-and-neck cancers, that results in irreversible damage of salivary glands. Loss of salivary fluid secretion leads to complications such as difficulty swallowing, rampant dental caries, oral mucosal lesions, and fungal infections that together severely affect the quality of life for patients [26,27]. This condition has been reproduced in several animal models, such as mouse, rats, mini-pigs, and non-human primates. Interestingly, irradiation (IR) induces considerable loss of saliva flow in the absence of extensive tissue damage or loss of acinar cells. While fibrosis and loss of tissue can occur, the onset and severity of this phase of cellular damage differs among the various species. Thus, the mechanism underlying IR-induced loss of salivary gland function is a subject of great interest in the field, with clinical studies being directed towards assessing therapies targeted to recovery of cell function, prevention of functional loss, or regrowth of salivary glands. In this review we will summarize the current knowledge regarding the role of TRP channels in salivary gland function and radiation-induced secretory dysfunction.

## 2. Historical Overview of Transient Receptor Potential (TRP) Channels

An extensive search for the molecular components of SOCE led to the identification of the transient receptor potential (TRP) superfamily of cation channels. These channels are expressed in a variety of organisms, including worms, flies, zebrafish, mice, and humans, and are broadly divided into two groups based on sequence and topological similarities. Group 1 TRPs consist of five subfamilies that bear strong homology to the founding member, *Drosophila* TRP [28]. Of these, the TRPC subfamily is most related to *Drosophila* TRP. Other subfamilies in the group include TRPV, TRPM, TRPA, and TRPN. The TRPN proteins are not found in mammals, although they are expressed in some vertebrates, such as zebrafish. The group 1 TRPs have six transmembrane segments, including a pore loop situated between the fifth and sixth transmembrane segments. TRPC, TRPM, and TRPN channels also contain a TRP domain, which follows the sixth transmembrane segment and is quite conserved between the channels. Apart from the TRPM channels, the other group 1 TRPs have multiple ankyrin repeats in the N-terminus. Three TRPM channel members, TRPM2, TRPM6, and TRPM7, are unique in that they have a pore as well as a catalytic functional domain and thus, are often referred to as chanzymes [29,30]. Group 2 TRPs consist of TRPP and TRPML channels, which share substantial sequence homology over the transmembrane segments and contain a large loop separating the first two transmembrane domains. The first TRPP and TRPML members were discovered as gene products mutated in autosomal dominant polycystic kidney disease (ADPKD) and mucolipidosis type IV (MLIV) respectively [31,32,33,34]. It should be noted that other TRP channels have also been associated with conditions of inflammation, cell damage, and disease. For example, TRPC5 and TRPC6 have been linked to the most common gastrointestinal obstruction disease in infants. TRPM2 have been suggested to underlie neurodegenerative disorders that cause movement disorders, whereas a mutation in TRPA1 was implicated in debilitating body pain. TRPV4 has been implicated to multiple channelopathies involving the musculo-skeletal system As such, it is not surprising that many members of the TRP superfamily are considered to be promising targets for the development of novel therapeutics [35,36,37,38,39,40].

TRPs are non-selective cation-channels which display variable calcium permeability. They, however, contribute to calcium signaling mechanisms and regulation of many physiological processes in a plethora of cell types. Almost all TRP channels, except TRPC subfamily members, have been reported to have sensory function. There is substantial evidence to show that regulation of TRP channels is polymodal and that they can mediate transduction of a wide variety of environmental stimuli including mechanical, thermal, or chemical stimuli [41]. A large group of TRP channels respond to thermal stimuli. While TRPV1 was the first heat-activated channel to be identified, to date, 10 thermoTRP channels with distinct range of thermoensitivity have been identified in mammals: TRPV (TRPV1, TRPV2, TRPV3, and TRPV4), TRPM (TRPM2, TRPM3, TRPM4, TRPM5, and TRPM8), and TRPA (TRPA1). In rodents, TRPV1, TRPV2, and TRPM3 are activated by noxious heat, while TRPV3, TRPV4, TRPM2, TRPM4, and TRPM5 are activated by warmth [42,43,44]. Channels such as TRPM8 [45,46,47,48,49] and TRPA1 [50] have been reported to be activated by cold stimuli. However, the thermal sensitivity of TRPA1 from both humans and rodents remains a subject of debate [51,52] due to contradictory observations. TRPA1 from mice was first reported to be activated by cold stimulation when heterologously expressed in cultured cells [50]. However, a later study contended that TRPA1 was not a temperature-sensitive channel [53]. Note that TRPA1 channel activity can be modulated by Ca^2+^, receptor stimulation, pH, and osmotic pressure, which may explain the apparent contradictory observations by different studies [51,54,55,56,57,58,59,60]. A peculiar feature of thermoTRP channels is that they can also be activated by non-thermal stimulation. For example, TRPV4 is activated by hypotonic and mechanical stimulation [61,62], while TRPV1 is activated by capsaicin, contained in chili pepper, and also by extracellular acidic stimulation [46,63]. TRPA1 is activated by various irritating chemical compounds contained in plants, as well as environmental irritants such as acrolein contained in exhaust gas and cigarette smoke [64]. It is interesting that the sensitivity for thermal activation of TRP channels can be modified by other factors such as reduction of cellular PIP_2_ levels [65,66,67,68]. The physiologic roles and activation mechanisms regulating thermoTRP channels have been summarized in several comprehensive reviews [69,70,71].

## 3. TRPC Channel Regulation and Function

The TRPC subfamily consists of seven members (TRPCs 1–7) that are divided into four subsets based on their amino acid homology: TRPC1, TRPC2, TRPC3/TRPC6/TRPC7, and TRPC4/TRPC5. All TRPC channels display channel activation in response to receptor-stimulated PIP_2_ hydrolysis and have six transmembrane domains with a pore-forming domain located between the fifth and sixth domains. These channels contain N-terminal ankyrin repeats, and in the C-terminus, a highly conserved TRP domain, several calmodulin (CaM)-binding domains, and a putative IP_3_R binding site [72,73,74]. TRPC channels show diverse tissue expression, physiological functions, and channel properties. Recent reviews have presented a general overview of the molecular components and mechanisms regulating SOCE [22,75], as well as overviews of the individual TRPC channels: TRPC1 [76], TRPC2 [77], TRPC3 [78], TRPC4 [79], TRPC5 [80], TRPC6 [81], and TRPC7 [82]. TRPC2 is a pseudogene in humans [83,84]. To date, almost all TRPC channels have been proposed as possible molecular components of channels mediating SOCE. However, data for some TRPCs are not very consistent. So far, the strongest evidence for the contribution of TRPC channels to SOCE has been provided for TRPC1 and TRPC4, whereas the contribution of TRPC3 to SOCE appears to be dependent on cell type and level of expression. TRPCs 5, 6, and 7 have been generally described to be store-independent, with a few exceptions.TRPC1 was the first mammalian TRPC channel to be cloned [83,84], and early studies established that it is activated by conditions resulting in store depletion and associated with the generation of a relatively Ca^2+^-selective cation current that was termed I_SOC_ (store-operated Ca^2+^ current; [85]) to differentiate it from I_CRAC_, the current generated by functional Orai1 [86]. TRPC1 has been reported to contribute to SOCE in a variety of cell types [87,88], although heterologous expression of the channel does not always result in enhancement of SOCE. Note that unlike with Orai1 or STIM1, TRPC channel contribution to SOCE is not seen in all cell types. Importantly, while TRPC1 clusters with and is activated by STIM1 following store-depletion, its function is also dependent on Orai1 channel activity [21]. It was shown that Orai1-mediated Ca^2+^ entry triggers recruitment of TRPC1 to the plasma membrane. Thus, TRPC1 and Orai1 form separate channels that are activated by STIM1 following neurotransmitter simulation of salivary gland cells and contribute to the [Ca^2+^]_i_ increase seen in stimulated cells. Orai1 is the first channel to be activated while recruitment and activation of TRPC1 leads to amplification and modulation of [Ca^2+^]_i_ increase that is induced by Orai1. However, since TRPC1 is activated by STIM1 following clustering of the two proteins within ER-PM-junctions, TRPC1 function is dependent on ER-Ca^2+^ depletion as well as Orai1 channel activity [21].

## 4. TRPC Channel Function in Exocrine Glands

As noted above, early studies established that Ca^2+^ influx is the primary determinant of sustained fluid secretion from salivary acinar cells [6]. It is now widely accepted that the primary mode of Ca^2+^ entry in acinar cells that is required for fluid secretion is mediated by SOCE. The main molecular components involved in SOCE in salivary gland acinar cells have now been identified as members of the transient receptor potential canonical (TRPC) family, TRPC1 and TRPC3. Both channels contribute to SOCE in dispersed acinar cell preparations, as well as cultured salivary gland cell lines [6,17,89]. Knockdown of endogenous TRPC1 significantly decreased SOCE in the human salivary gland (HSG) cell line, as well as primary cultures of mouse pancreatic and submandibular gland cells [17]. Further conclusive evidence was provided by studies with mice lacking TRPC1 (TRPC1^−/−^), which showed reduced SOCE in salivary gland and pancreatic acinar cells as well as attenuation of Ca^2+^-dependent physiological functions [17,18], despite having normal viability, development, and behavior [81]. SOCE is fundamentally important for fluid secretion in salivary glands and for protein secretion in the exocrine pancreas. TRPC1^−/−^ mice displayed reduction in salivary gland fluid secretion that was associated with a decrease in SOCE and K_Ca_ activity in acinar cells from the mice [18,89]. Similarly defects in Ca^2+^-activated Cl^−^ channel activity and protein secretion, as a consequence of reduced SOCE, were reported in pancreatic acinar cells [17]. Notably, while there is no change in Orai1 in salivary gland and pancreatic acinar cells from TRPC1^−/−^ mice, the channel does not appear to compensate for the lack of TRPC1 or support cell function on its own. Hence, decreased secretory function in these exocrine glands is primarily due to the loss of TRPC1-mediated SOCE. The caveolae-residing protein, caveolin-1 (Cav-1), is an important modulator of TRPC1 activity and functions as a plasma membrane scaffold for the channel. In the absence of Cav-1, TRPC1 is mislocalized and unable to interact with STIM1 [90]. Consistent with this, localization of TRPC1, its interaction with STIM1, as well as SOCE were disrupted in salivary gland acinar cells from Cav-1^−/−^ mice [91]. These cellular defects were associated with reduced fluid secretion in the mice. Together, these findings establish a vital role for TRPC1 in salivary gland fluid secretion.

TRPC3 is reported to contribute to both the store-operated and receptor-activated calcium entry pathways, and has been associated with the generation of a non-selective, Ca^2+^-permeable channel in response to receptor-stimulated PIP_2_ hydrolysis. While the channel can be directly activated by application of diacylglycerol to cells, it also contributes to SOCE under some conditions. Mice lacking TRPC3 show reduced SOCE and fluid secretion [92]. Interestingly, the contribution of TRPC3 to SOCE is dependent on the presence of TRPC1, as TRPC1^−/−^ mice do not display TRPC3-dependent SOCE [93]. Thus, it has been proposed that either the channels are assembled as a store-operated heteromeric channel, or that TRPC1 is required for store-dependent regulation of TRPC3. Indeed, TRPC3–TRPC1 interaction is necessary for STIM1 regulation of the channels in salivary gland ductal cells. The two TRPC channels coimmunoprecipitate following cell stimulation together with STIM1. Loss of TRPC1 eliminates the association of STIM1 with TRPC3 [93,94]. TRPC3-mediated Ca^2+^ entry can also contribute to exocrine gland pathology and tissue damage. Pancreatic acini from TRPC3^−/−^ mice showed significant protection from acute pancreatitis induced by hyper-activation of SOCE. Similar effects were seen by blocking channel function in TRPC3^+/+^ mice by treatment with pyrazole 3, a TRPC3 channel inhibitor [92,95].

Orai1 is a critical and essential component of SOCE [10,12]. Although the role of Orai1 in salivary gland function is yet to be determined, it has been examined in two other exocrine glands, lacrimal and pancreatic. Orai1^−/−^ mice display loss of lacrimal gland function and reduced SOCE in lacrimal gland acinar cells [96]. Further, knockdown of Orai1 in isolated pancreatic ductal cells also resulted in loss of SOCE and Ca^2+^-activated ion channel activity that was similar to that seen in TRPC1^−/−^ cells [17]. Targeted knockout of Orai1 in pancreatic acinar cells of adult mice led to loss of SOCE and severely compromised pancreatic secretion. Antimicrobials secreted by pancreatic acini play an important role in shaping the gut microbiome, as well as maintaining the innate immunity and barrier function in the intestines [97]. Mice lacking acinar Orai1 exhibited intestinal bacterial outgrowth and dysbiosis, ultimately causing systemic translocation, inflammation, and death.

## 5. TRPV4 and Other TRP Channel Function in Salivary Glands

Regulation of cell volume in response to changes in osmolarity is critical in salivary gland fluid secretion. In response to carbachol (CCh) stimulation, cells undergo a decrease in cell volume, which then recovers via a regulatory volume increase (RVI). Conversely, hypotonic conditions lead to cell swelling and volume recovery via regulatory volume decrease (RVD). Both these processes depend on the water permeability of the cells, which in salivary gland cells is determined by the level of AQP5 in the membrane. A role for TRPV4 in RVD was previously reported by an earlier study reported by us [98]. TRPV4 was activated by cell swelling under hypoosmotic conditions and that Ca^2+^ entry via TRPV4 was important for regulating the ion fluxes involved in driving RVD. This study demonstrated a novel association between osmosensing TRPV4 and AQP5. Acinar cells from mice lacking either TRPV4 or AQP5 displayed greatly reduced Ca^2+^ entry and loss of RVD in response to hypotonicity, although the extent of cell swelling was similar. Recent studies have shown a more direct role for TRPV4 in fluid secretion. TRPV4 is activated by endogenous arachidonic acid metabolites, 4α-phorbol-12,13 didecanoate, GSK1016790A, moderate heat, and mechanical stress. Pharmacological TRPV4 activation using the selective agonist GSK1016790A caused Ca^2+^ influx in isolated acinar cells in a basal-to-apical wave. Consistent with these observations, GSK1016790A elicited salivation in the perfused submandibular gland that was dependent on extracellular Ca^2+^ [99]. Another study reported a functional interaction between TRPV4 and the Ca^2+^-activated chloride channel, ANO1, in acinar cells isolated from mouse salivary and lacrimal glands [100]. Activation of TRPV4 induced an increase in fluid secretion, ANO1 activation and a volume decrease in acinar cells by increasing [Ca^2+^]_i_. Muscarinic stimulation of saliva and tear secretion was downregulated in both TRPV4-deficient mice and in acinar cells treated with a TRPV4-specific antagonist (HC-067047). Furthermore, the temperature dependence of muscarinic salivation was shown to depend mainly on TRPV4. This study also showed a novel association between TRPV4, IP_3_Rs, and ANO1 that collectively contributes to the regulation of salivation and lacrimation.

Additional TRP channels have also been identified in salivary glands. Immunohistochemistry has revealed the presence of TRPM8, TRPA1, TRPV1, TRPV3, and TRPV4 in myoepithelial, acinar, and ductal cells of the sublingual, submandibular, and parotid glands. Interestingly, perfusion of the entire submandibular gland with the TRPV1 agonist capsaicin (1 μM) via the submandibular artery significantly increased CCh-induced salivation, whereas perfusion with TRPM8 and TRPA1 agonists (0.5 μM WS12 and 100 μM allyl isothiocyanate) decreased it. Application of agonists for each of the thermosensitive TRP channels increased [Ca^2+^]_i_ in a cultured submandibular epithelial cell line. These results indicate that temperature-sensitive TRP channels are localized and distributed in acinar, ductal and myoepithelial cells of salivary glands, and that they might have a functional role in regulating and/or modulating saliva secretion. Further studies will be needed to characterize the exact role of temperature-dependent regulation of salivary gland function and the involvement of TRP channels in this mechanism.

## 6. Role of TRPM2 in Salivary Gland Dysfunction

### 6.1. Regulation and Activation of TRPM2

TRPM2 is the second member of the TRPM subfamily, which includes eight functionally diverse members, namely TRPM 1–8. TRPM2 (previously known as LTRPC2 or TRPC7) is a Ca^2+^ permeable, non-selective cation channel. It is predominantly expressed in the brain and has also been detected in bone marrow, spleen, heart, liver, lung and immunocytes, salivary gland [101,102,103]. TRPM2 is unique in that its structure contains a Ca^2+^-permeable non-selective cationic pore fused to an enzyme of the Nudix family of pyrophosphatases. Adenosine diphosphate ribose (ADPR) is considered the primary gating molecule of TRPM2 [104]. The channel displays a linear current-voltage (I–V) relationship, and substantial permeation to cations such as Na^+^, K^+^, Ca^2+^, Mg^2+^, and Zn^2+^, with relative permeabilities of P_K_/P_Na_ ∼ 1.1, P_Ca_/P_Na_ ∼ 0.9, and P_Mg_/P_Na_ ∼ 0.5. Most importantly, TRPM2 serves as a sensor for reactive oxygen species (ROS) in cells, since increase in cellular ROS or nitrogen species, cause formation ADPR. Intracellular Ca^2+^ also facilitates TRPM2 activation by enhancing the channel sensitivity to ADPR [101].

Under oxidative stress, ADPR formation is mediated through activation of the PARP/PARG (Poly(ADP-ribose) polymerase/glycohydrolase) pathway in the nucleus. ADPR is also synthesized in the mitochondria, which contain the largest pool of intracellular nicotinamine adenosine diphosphate (NAD+), and is released into the cytosol [105,106]. Convincing evidence has been presented to show that ROS-induced TRPM2 activation is also triggered via the production of ADPR from mitochondria [107,108]. It is now clearly established that TRPM2 channel serves as an important pathway for oxidative stress-induced increases in [Ca^2+^]_i_, which regulate Ca^2+^ signaling mechanisms that include regulation of ion channel activities, gene expression, secretion, apoptosis, and inflammasome assembly. TRPM2 also responds to warm temperatures that act synergistically with ADPR, NAD^+^, and cADPR at concentrations that otherwise cannot activate the channel [101,102,105,106,109]. In normal physiological states, a major function of TRPM2 is to modulate the immune system by controlling cytokine release in human monocytes, including tumor necrosis factor-alpha (TNFα), interleukin 6 (IL-6), IL-8, and IL-10, and the maturation and chemotaxis of dendritic cells. Non-physiological stimulation of TRPM2 is suggested to lead to pathology and dysfunction.

### 6.2. TRPM2 and Radiation-Induced Loss of Salivary Flow

**Figure 2:**
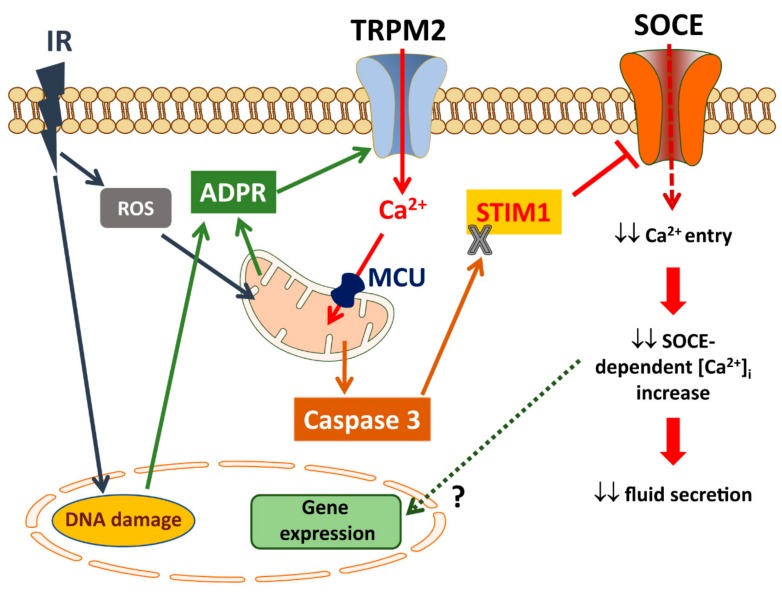
Role of TRPM2 in radiation -induced salivary gland dysfunction. See text for description.

A debilitating side effect of radiation treatment in patients with head and neck cancers is xerostomia, or dry mouth, as a result of severe decrease in saliva secretion. While acute effects of IR could be induced by membrane/protein damage, the more delayed and long-term effects have been proposed to be caused by damage of progenitor cells within the adult salivary gland [110,111,112]. However, the decrease in saliva secretion cannot be strictly correlated with a decrease in acinar cells or damage of the gland. In fact, in mouse models of radiation, glandular loss, and fibrosis are not seen for about four-to six months after radiation while loss of function is almost immediate and persists even after the radiation-induced ROS in the tissue has been cleared [113,114]. Our recent studies demonstrate a critical role for TRPM2 in radiation-induced persistent loss of salivary gland fluid secretion (Figure 2). TRPM2 is present in salivary gland acinar cells and is activated under conditions which increase ROS, such as by treatment with H_2_O_2_ or following radiation treatment of the salivary glands in mice [103]. Importantly, while TRPM2^+/+^ mice display persistent loss of salivary gland fluid secretion that is detected within 10 days after radiation, mice lacking TRPM2 (TRPM2^−/−^) demonstrate transient loss of function with >80% recovery of function by 30 days after IR. Activation of TRPM2 by radiation has been supported by data showing that Ca^2+^ influx is constitutively activated in acinar cells isolated from TRPM2^+/+^ mice 24 h after radiation. This increase in plasma membrane Ca^2+^ permeability is not seen in acini from irradiated TRPM2^−/−^ mice. Treatment of mice with the PARP1 inhibitor, 3-AB, prior to radiation suppresses TRPM2 activation and exerts protection of salivary gland function. Further, TEMPOL, a redox-cycling nitroxide and ROS scavenger that has been reported to protect several organs, including the heart and brain, from ischemia/reperfusion damage [115], also protects salivary gland function in irradiated mice [103,114]. Thus, the presence of TRPM2 in acinar cells converts an inherently reversible loss of salivary gland function, following radiation treatment to an irreversible one.

In searching for the mechanism(s) linking early activation of TRPM2 by radiation to the persistent loss of salivary fluid secretion, we have now demonstrated that persistent reduction in STIM1 protein, and SOCE, underlies radiation-induced loss of salivary gland function. Furthermore, the decrease in STIM1 protein levels is linked to activation of TRPM2 [113]. A major finding of this study was that TRPM2-mediated increase in [Ca^2+^]_i_ in response to radiation causes an increase in mitochondrial [Ca^2+^] and ROS_mt_ but a decrease in mitochondrial membrane potential. This is accompanied by a relatively slower appearance of activated caspase-3 which persists for about a month after the treatment. In irradiated TRPM2^−/−^ mice, the increases in [Ca^2+^]_mt_, ROS_mt_ and activated caspase-3 are substantially attenuated. Importantly, TRPM2-dependent activation of caspase-3 is correlated with loss of STIM1. These interesting findings reveal that radiation-induced loss of salivary gland fluid secretion is mediated via a TRPM2-dependent pathway that impacts mitochondrial function and leads to irreversible loss of SOCE. Notably, cleavage of STIM1 by calpain and γ-secretase has been associated with stress and Alzheimer’s disease, respectively [116,117]. Further, proteasome inhibition reduces SOCE by promoting autophagy-mediated degradation of STIM1/2 [118]. Future studies will need to clarify exactly how the long-term suppression of STIM1 expression is controlled. Most likely, remodeling of gene expression or other epigenetic changes occurring in irradiated salivary glands might be involved. Ca^2+^ entry mediated by SOCE, via Orai1 channels, is critical for the regulation and activation of transcription factors such as NFAT and cFos, as well as other channels that regulate NFκB (e.g., TRPC1). Attenuation of these signaling mechanisms due to loss of channel activation could impact the expression of STIM1 or other cellular proteins, further depressing the occurrence of downstream events that are triggered by these signaling events. It is also important to consider that loss of STIM1 and SOCE can potentially affect other cellular processes, including regeneration of salivary gland cells [119]. In a salivary gland cell line, silencing the mitochondrial Ca^2+^ uniporter or caspase-3, or treatment with inhibitors of TRPM2 or caspase-3 prevented irradiation-induced loss of STIM1 and SOCE. Importantly, expression of exogenous STIM1 in the salivary glands of irradiated mice increases SOCE and fluid secretion. Thus, targeting the mechanisms underlying the loss of STIM1 would be a potentially useful approach for preserving salivary gland function after radiation therapy [113].

### 6.3. TRPM2 in Inflammatory Disorders

Oxidative stress plays a critical role in various pathophysiological processes, including cancer, acute and chronic neurodegenerative disorders (Alzheimer’s and Parkinson’s diseases); diabetes mellitus, atherosclerosis, ischemia/reperfusion injury, and autoimmune disease; and in normal cellular functions [120]. Main pro-inflammatory molecules present in chronic inflammatory responses are ROS, reactive nitrogen species (RNS), IL-2, IL-4, IL-5, IL-7, IL-13, IL-9, IL-10, IL-12, IL-17, IL-21, interferon (IFN)-γ, transforming growth factor (TGF)-β, and tumor necrosis factor (TNF)-α. Salivary gland epithelial cells themselves synthesize and secrete cytokines to maintain barrier protection and regulate anti-inflammatory processes. Furthermore, immune cells produce ADPR via CD38 and CD157 signaling or by activating the PARP pathway, both of which can facilitate activation of TRPM2. Although there are no data available presently that demonstrate a role for TRPM2 in inflammatory disorders of the salivary gland, the available information suggests that the channel could be activated in response to inflammatory conditions and contribute to the pathogenesis.

There is increasing evidence for the involvement of TRPM2 in innate immunity, inflammation, regulation of cytokine production, cellular migration and ROS production [105]. NADPH oxidase-dependent ROS production in phagocytic cells is triggered in response to infection and plays a key role in inflammation. Activation, migration as well as regulation of the effector mechanisms of immune cells critically depend on Ca^2+^-entry into the cell. While Orai1 channels also mediate this type of calcium influx to regulate the function of T cells and B cells, TRPM2 can also contribute to the elevation of [Ca^2+^]_i_ as it is expressed in the plasma membrane of neutrophils, T and B lymphocytes, and dendritic cells. In T cells, cross-linking of cell surface receptors induces a rise of ADPR endogenously generated from NAD^+^ which can activate TRPM2 [121]. Importantly, inhibition of NAADP signaling in T cells [122] reduces antigen-induced proliferation and cytokine production and ameliorates clinical symptoms of experimental autoimmune encephalomyelitis (EAE, [122]). The role for TRPM2 in lymphocyte function is now been widely accepted. It has been shown that TRPM2-mediated Ca^2+^ influx regulates T cell proliferation and proinflammatory cytokine secretion following polyclonal T cell receptor stimulation. TRPM2-deficiency or treatment with TRPM2 channel blockers significantly modulate effector T cell function [123,124]. Moreover, TRPM2 channels impact the maturation and chemokine-activated directional migration of dendritic cells, which function as antigen-presenting cells [125]. TRPM2 can also be activated by triggering of toll-like receptors by LPS and cytokine receptors (TNFα) as well as by intracellular ADPR. It is suggested that inhibition of TRPM2 channels in autoimmune inflammatory disorders will likely dampen the adaptive T cell-mediated immune response without favoring prolonged T cell survival and inflammatory tissue damage.

On the other hand, it has been reported that TRPM2-mediated Ca^2+^ influx controls the ROS-induced signaling cascade responsible for chemokine production, which aggravates inflammation [126]. TRPM2 expressed in macrophages and microglia aggravates peripheral and spinal pro-nociceptive inflammatory responses, and contributes to the pathogenesis of inflammatory and neuropathic pain [127]. TRPM2 critically influences T cell proliferation and proinflammatory cytokine secretion following polyclonal T cell receptor stimulation. Consistently, TRPM2-deficient mice exhibit an attenuated clinical phenotype of EAE with reduced inflammatory and demyelinating spinal cord lesions [128]. In addition, TRPM2 regulates macrophage polarization and gastric inflammation during *Helicobacter pylori* infection [129]. TRPM2 channels mediate bleomycin-induced lung inflammation in alveolar epithelial cells [130], and contributes to antigen-stimulated Ca^2+^ influx in mucosal mast cells [131].

Numerous proinflammatory cytokines are produced during the innate immune response to infection and inflammation, several of which have been linked with activation of TRPM2. Knockdown of TRPM2 attenuates LPS-induced production of IL-6, IL-8, IL-10, and TNF-α in THP1 monocytic cells. The corresponding decrease in LPS-induced Ca^2+^ influx under these conditions supports the suggestion that TRPM2-mediated Ca^2+^ influx has a significant role in generating these cytokines. Zymosan-induced production of granulocyte colony-stimulating factor (G-CSF) and IL-1α was also strongly attenuated in macrophages from the TRPM2^−/−^ mice. Sulfur mustard (SM), an alkylating agent used in chemical warfare, causes tissue damage and induces inflammatory responses. SM-induced production of IL-6, IL-8, and TNF-α by human neutrophils requires TRPM2-mediated Ca^2+^ influx to activate the p38 mitogen-activated protein kinase (p38 MAPK) signaling pathway [132]. The production of IL-6 and TNF-α was however enhanced in LPS-treated macrophages from the TRPM2^−/−^ mice and in response to LPS-induced infection in these mice [133]. Evidently, further studies are required to clarify the noticeable discrepancies from these studies that used different infection stimuli and cell preparations. The production of IL-12 and IFN-γ after dextran sulfate sodium-induced colon inflammation is significantly decreased in the TRPM2^−/−^ mice [126]. Further analysis suggests that the TRPM2 channel function is required for the production of IL-12, the early inflammatory cytokine produced by dendritic cells and possibly other immune cells as well, which elicits IFN-γ-mediated innate immune responses. The deficient production of IL-12 and IFN-γ in the TRPM2^−/−^ mice led to a significantly lower survival rate after *Listeria monocytogenes* infection, supporting a vital role for the TRPM2 channel in the innate immune response to this infection [134]. A recent study shows that LPS/IFN-γ-induced increase in the [Ca^2+^]_i_ and subsequent release of nitric oxide in microglia also depends on the TRPM2 channel function [135]. Immune cells such as macrophages and microglia also produce IL-1β, a key proinflammatory cytokine in innate immunity [136]. The priming signal stimulates a Toll-like receptor (TLR) such as TLR4 by LPS or other receptors to initiate signaling pathways leading to synthesis of pro-IL-1β. TRPM2 channels mediate Ca^2+^ influx as the major ROS-induced Ca^2+^ signaling mechanism in macrophages [137], which regulates NLRP3 inflammasome activation in macrophages by particulates such as charged lipids, silica, and alum. This process is impaired in macrophages from the TRPM2^−/−^ mice [138]. Thus, TRPM2-mediated Ca^2+^ influx is a critical step in coupling ROS generation to NLRP3 inflammasome activation and IL-1β maturation.

Notably, Sjøgren’s Syndrome (SS) has been associated with overexpression of proinflammatory cytokines, including TNF-α, IL-7, IL-1β, IL-6, IL-10, IL-17, IL-18 and gamma-interferon (γ-IFN) [139,140,141]. Moreover, one of these cytokines, IL-6, was correlated with poor quality of life in SS patients [140]. Based on a body of evidence related to different pathological conditions, TNF-α and its interactors are recognized to be involved in a pro-inflammatory/pro-oxidant condition, implicating the relevance of redox imbalances in SS pathogenesis [142,143]. Related to excess expression of proinflammatory cytokines, a pro-oxidant state could be postulated in SS based on the established evidence for a mechanistic association of a pro-inflammatory condition and oxidative stress in a number of disorders including, e.g., cancer, cardiovascular, neurological and pulmonary diseases, and diabetes [143,144,145,146]. Furthermore, it should be noted that ROS is also produced by activated granulocytes during inflammation both in SS pathogenesis and in other systemic disorders with autoimmune features (e.g., systemic sclerosis). Cejková et al. has reported that *Trpm2* knockout mice showed attenuation of inflammatory indicators such as production of CXCL2, neutrophil infiltration and ulceration [147]. Thus, ROS-evoked Ca^2+^ influx via TRPM2 could represent a key inflammatory mediator in monocytes and in the epithelium of both salivary and lacrimal glands of SS patients. It will be very important to investigate the role for this channel in SS-induced salivary gland pathology. Establishing this will provide new strategies for treatment of the disease.

## 7. Conclusions

Studies done over the past 30 years have provided a tremendous amount of information about the key molecular components that regulate salivary gland fluid secretion, including those involved in Ca^2+^ signaling, ion transport, and water transport. Future studies should be focused on establishing the mechanisms underlying salivary gland dysfunction. Such studies should provide novel targets and strategies for treatment. On such target in salivary glands is TRPM2, which appears to be critically involved in radiation-induced irreversible loss of salivary gland fluid secretion. Based on data reported by us and others, potential therapeutic strategies could include manipulating channel activity, developing specific inhibitors of the channel or TRPM2-dependent signal transduction cascade. In the case of radiation-induced salivary gland dysfunction, we propose that inhibitors of TRPM2 or caspase-3, scavengers of ROS in the cytosol or mitochondria, as well as inhibitors of PARP1 could be used to protect against loss of function. Additionally, its ability to respond to ROS has made TRPM2 a potential therapeutic target for chronic inflammation and neurodegenerative diseases. TRPM2 ion channel and its gating molecule ADPR are previously unsuspected players necessary for robust cytokine production and innate cell activation during intracellular bacterial infection. These findings highlight the potential of the metabolic manipulation of ADPR levels or modulating TRPM2 activation modalities to exert immunomodulation. However, currently, direct evidence for TRPM2 involvement in Sjøgren’s Syndrome (SS) is lacking. Based on currently available data highlighting the role of TRPM2 in inflammatory process, it will be very important to assess whether TRPM2 contributes to Sjøgren’s Syndrome (SS). Identification of the role of other, including sensory, TRP channels in salivary gland function will also provide additional targets for modulating water secretion from the gland. Future studies should focus on these potentially novel and important roles of these TRP channels.
